# Next-generation probes, particles, and proteins for neural interfacing

**DOI:** 10.1126/sciadv.1601649

**Published:** 2017-06-09

**Authors:** Jonathan Rivnay, Huiliang Wang, Lief Fenno, Karl Deisseroth, George G. Malliaras

**Affiliations:** 1 Department of Biomedical Engineering, Northwestern University, Evanston, IL 60208, USA.; 2 Palo Alto Research Center, Palo Alto, CA 94304, USA.; 3 Departments of Bioengineering and Psychiatry, Stanford University, Stanford, CA 94305, USA.; 4 Department of Bioelectronics, École Nationale Supérieure des Mines, CMP-EMSE, MOC, Gardanne 13541, France.

**Keywords:** Neural interface

## Abstract

Bidirectional interfacing with the nervous system enables neuroscience research, diagnosis, and therapy. This two-way communication allows us to monitor the state of the brain and its composite networks and cells as well as to influence them to treat disease or repair/restore sensory or motor function. To provide the most stable and effective interface, the tools of the trade must bridge the soft, ion-rich, and evolving nature of neural tissue with the largely rigid, static realm of microelectronics and medical instruments that allow for readout, analysis, and/or control. In this Review, we describe how the understanding of neural signaling and material-tissue interactions has fueled the expansion of the available tool set. New probe architectures and materials, nanoparticles, dyes, and designer genetically encoded proteins push the limits of recording and stimulation lifetime, localization, and specificity, blurring the boundary between living tissue and engineered tools. Understanding these approaches, their modality, and the role of cross-disciplinary development will support new neurotherapies and prostheses and provide neuroscientists and neurologists with unprecedented access to the brain.

## Teaser

Multimodal and multidisciplinary approaches lead to next-generation technologies for reading and modulating neural function.

## INTRODUCTION

Luigi Galvani’s experiments linking electricity with motor activity laid the foundation for current knowledge of signaling in the nervous system as analogous to circuits in modern computer processors. The refined use of electricity in neuroscience, usually with electrodes, has furthered our knowledge of how the brain collects sensory input from the environment, processes this information in the context of experience, and controls the rest of the body in response. Electrodes have become ever more refined in their application as readout and control devices, being now packed into small arrays able to be chronically implanted into salient cortical regions and to observe activity patterns of hundreds of neurons during behavior. Therapeutically, there are established and safe interventions to interrupt or stimulate stereotactically defined targets in patients with Parkinson’s disease (*1*) or essential tremor (*2*), and there are clinical trials for obsessive compulsive disorder (*3*) and major depressive disorder (*4*). Moreover, electrodes and arrays have seen impressive closed-loop applications for patients with spinal cord injury (*5*).

Neurologic disorders account for 7% of total global burden of disease measured in disability-adjusted life years, with just under half of this sum attributed to neuropsychiatric disorders (including Alzheimer’s disease, Parkinson’s disease, and epilepsy), and the rest to cerebrovascular diseases (that is, stroke) (*6*, *7*). The social and economic burden of these diseases has motivated and continues to motivate technological advance and development in neuroengineering, medicine, and science. To date, these tools, combined with pharmacology, have been the workhorse of interventional and observational neuroscience research. The past decade, in particular, has seen an explosion in neuroscience research, driven by improved methods and devices, and by the development, distribution, and creative application of novel neuromodulatory and observational tools that have allowed for cell type–specific manipulation in model organisms. These developments have been recognized and stimulated by immense initiatives and funding programs. One example is the United States’ BRAIN Initiative (*8*), which exists to “accelerate the development and application of new technologies that will enable researchers to produce dynamic pictures of the brain that show how individual brain cells and complex neural circuits interact at the speed of thought,” in part to facilitate “progress in diagnosing, treating, and potentially curing the neurological diseases and disorders that devastate so many lives.” The Human Brain Project (*9*) is a distinct transnational and ambitious effort in Europe to develop informatics and communication infrastructure for neuroscience and to further brain-inspired computing.

Here, we review current efforts aimed to move beyond the limitations of traditional electrode-based recording and intervention protocols. Many of the newer approaches are limited to use in experimental settings, but some early results from the laboratory have shown promise toward translation to clinical settings. We provide an overview of these translational approaches and comment on possible future directions to further improve the link between bench and bedside.

The central nervous system constantly receives sensory information, processes the stimuli, assigns significance based on past experiences, and decides on a course of action that is carried out through neural signaling—for example, by increasing blood pressure or heart rate, controlling movement via muscles, or altering internal processing as with savoring a taste of food and allowing the mind to wander. In working to create better and more effective modalities for clinical neuroscience, it is important to seek understanding of how the myriad neurons of the brain work together to go from sensation to thought to action and to identify key causal components of these distributed neural networks. In this way, it may be possible for dysfunctional tissue to be bypassed through sensing of upstream neural activity and delivery of artificial downstream signals (*5*).

The membrane potential of an individual neuron rests at approximately −70 mV. This potential will fluctuate with excitatory (depolarizing) and inhibitory (hyperpolarizing) inputs from other neurons. Given sufficient net excitatory input, an action potential will be generated, and the neuron will “fire”: The membrane potential will surpass a threshold (~−55 mV), causing the opening of voltage-gated channels that flood the neuron with positively charged sodium ions, resulting in rapid depolarization. Upon reaching a potential of +30 to 40 mV, the membrane repolarizes via the expulsion of potassium ions and relaxes back to its resting state. This impulse is propagated down the length of the axon until reaching the synapse, where voltage-gated calcium channels open, subsequently causing vesicles filled with neurotransmitters to release their cargo into the synaptic cleft between the axon and its downstream partner dendrite. These neurotransmitters can have either excitatory or inhibitory function on the downstream neuron. Thus, this process of information transfer translates electrical signals into chemical signals and then back again as the process repeats, propagating/modifying the initial signal. On a larger scale, the generation of action potentials by ensembles of neurons may be entrained with one another to create oscillations and rhythms that give rise to local field potentials (LFPs) (*10*), and these then may act to coordinate activity across even larger brain regions, influencing brain-wide activity and thus behavior.

In taking aim at neurological or psychiatric disease, understanding salient sample size and scale is of critical importance in designing and implementing readout and control devices. The implements of the trade thus include devices and tools capable of neural activity readout at different levels of resolution as well as control modalities that again range from single, defined types of neurons to regional modulators. The need to connect the realm of microelectronics, optics, and medical instrumentation with the soft, ever-evolving circuitry of the brain poses significant challenges. Bridging this inherent mismatch requires us to understand the interaction of these physical tools with living tissue, to manipulate the existing machinery of the cells themselves, and to find new ways to relay information into and out of the brain. The journey to understand how the mind works has advanced hand-in-hand with the application of these tools, and the two benefit from each other enormously, allowing researchers to answer previously unanswerable questions.

In this Review, we discuss recent efforts toward bidirectional neural interfacing based on engineered probes and their evolving materials and form factors, as well as micro- and nanoparticles, molecules, and proteins for localized and specific stimulation and recording. These advancements are roughly grouped by modality, starting with electrical interfacing, and building toward optical, magnetic, and other means of recording or stimulation, including exciting developments in genetically engineered protein neuronal activity indicators, as well as light-activated ion pumps and channels (that is, optogenetics). We address the use of multiple modalities in series and in parallel and conclude with an outlook discussing the current needs and existing hurdles. By bringing together efforts spanning electronics and mechanics through genetics and molecular biology, we hope to highlight the multidisciplinary efforts necessary to bring new tools to neuroscience, neuroengineering, and neurosurgery.

## ELECTRICAL RECORDING AND STIMULATION

Historically, electrodes have been the most commonly used conduit through which the signals of the brain are interrogated for research purposes as well as for diagnostic and for therapeutic applications. Communication between cells in the nervous system is dependent on ion fluxes, which can be recorded as electrical potentials; conversely, these cells can be stimulated through injection of electrical current, allowing for bidirectional electrical interfacing.

The neural signals recorded by most implanted electrodes are changes in the extracellular field due to ion fluxes in the local environment, allowing for recording of population activity in the form of LFPs (<~350 Hz), and, in some cases, the spiking activity or action potentials of individual neurons (~kHz). The potential at a recoding site depends on the magnitude of the nearby events, their polarity, and the distance from the recording site (*10*–*12*).

**Fig. 1:**
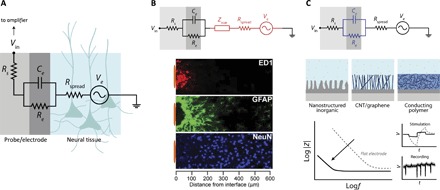
Electrical interface in neural tissue. (**A**) Equivalent circuit of electrode/tissue interface; in this case, recording is considered [that is, neurons acting as a voltage source (*V*_e_) and use of an amplifier]. However, similar concepts apply for stimulation. (**B**) Influence of neuroinflammatory reaction (astroglial scar) on local electrical impedance. The neuroinflammatory response adversely affects the signal from the neurons and the spreading resistance and introduces a scar impedance (*Z*_scar_) due to the formation of a dense layer of inflammatory cells (ED1), astrocytes (GFAP), and a distancing of neurons (NeuN) from the recording site. Fluorescence image is reproduced, in part, from the study of Biran *et al*. (*31*). (**C**) Influence of enhanced electrode coatings on improving the impedance of the electrode itself. Nanostructuring of traditional electrode materials, use of CNTs/graphene, or conducting polymers (CPs) allow for intimate ion interaction with the electrode, allowing for a marked drop in impedance. The comparison of impedance and resulting stimulation profile for a given biphasic current pulse and recording quality [signal-to-noise ratio (SNR)] is shown for a flat electrode (gray, dotted) and for an electrode with an enhancing coating (black line; for example, CPs).

The ability to record these physiological and pathological signals or to electrically stimulate a population of neurons depends on the impedance—the resistance to current flow—between the cell/tissue and the recording or stimulating device. Application and materials constraints, as well as availability and proximity of analog signal amplifiers, inform or ease the requirements for electrode impedance. The effective impedance can often be modeled, optimized, and understood through the use of equivalent circuit models, such as the one shown in Fig. 1A. In this case, the signaling in the neural tissue can be thought of as a low-impedance voltage source. *R*_spread_, sometimes referred to as *R*_media_, describes the resistance of the extracellular space and depends on the geometry of the recording site. *R*_e_ and *C*_e_ are attributed to the electrode itself—the leakage resistance and electrical double-layer capacitance of the electrode/tissue interface, in the simplest case. The electrode impedance is often modeled using constant phase elements, Warburg impedances, or transfer line models depending on the nature of the said interface, described below. Finally, *R*_s_ is the resistance of the interconnects that leads to higher-level circuitry such as amplifiers; *R*_s_ is often negligible in the case of metallic interconnects but is significant where organic conductors are used to transmit signals (*11*, *13*). All other aspects being equal, a lower impedance interface allows one to more readily “see” the voltage source that is the neural activity.

Similar to recording, electrical stimulation is also enhanced with a lower interface impedance, allowing for a higher charge injection limit and thus more efficient and safer stimulation with micrometer-scale electrodes. Governed by the same equivalent circuit, a stimulation waveform (typically a millisecond-scale biphasic current pulse) leads to a transient voltage output consisting of a rapid step, attributed to *R*_spread_, and a capacitive charging (inversely proportional to *C*_e_) (*14*). A low *C*_e_ leads to a large potential drop at the electrode/tissue interface, which can lead to electrolysis of water, electrode degradation, and/or tissue damage. Thus, efforts to minimize impedance are beneficial to both electrical recording and stimulation.

The nature of electrical recording ultimately depends on the application of interest (*11*, *15*). Electroencephalography (EEG), for example, is the least invasive implementation of electrodes, where large electrodes probe the potential, summed over a large population of neurons and attenuated by the skin/skull; the entire regions of the brain are collectively probed to investigate communication within the brain and rhythms arising from specific stimuli or states of consciousness. Its noninvasiveness makes this method a routine tool in clinical settings, where the main challenges include the need to minimize the electrical impedance between the skin and the recording surface. Conformal skin-like form factors, which follow the curvilinear morphology of scalp, have been targeted (*16*), as well as compliant, dry electrodes (*17*), to minimize the need for gel intermediaries that dry out after a short use period. More invasive approaches, such as epidural interfacing, or recent efforts to introduce stimulation/recording electrodes into cortical veins aboard small stents (stentrode) (*18*) represent exciting avenues for electrical interfacing without breaching the blood-brain barrier. However, the need to directly interface with neural tissue is motivated by multiple areas of neuroscience and neuroengineering. For instance, electrical measurement of a single neuron activity is thought to be achievable only with implanted electrodes nearby to firing cells. Unit activity allows us to better understand the low frequency rhythms (*10*), to map and understand the wiring of the brain and its link to perception, motion, and memory. Implanted devices can be used for localizing epileptogenic zones and for treating symptoms of Parkinson’s disease, among others, and are thought to yield the most useful control signals for brain-machine interfacing applications (*19*).

### Advancing the state of the art in implanted electrodes

The principles guiding implantable, electrode-based devices are general for both arrays on the surface of the brain (subdural) and probes/stimulators that penetrate into the tissue. An electrode site must be able to record or stimulate the same, intended, population of cells over a sufficiently long duration while causing minimal damage to tissue and eliciting minimal immune response. Early implanted electrodes relied on insulated metallic microwires or cone electrodes (*13*). The rapid developments in the microelectronics industry subsequently opened up the realm of rigid, patterned, and micromachined probes (*20*) such as the Michigan-style probes (*21*) and Utah arrays (*22*), which are considered as today’s state of the art in commercially available tools for neuroscience research. Through their many successes, enabling many groundbreaking discoveries in neuroscience (from the discovery of place and grid cells to mapping and stimulation of the motor cortex), implanted electrodes face numerous barriers that limit their broad implementation. Their rigid nature often leads to device encapsulation and degradation of their recording/stimulating capacity due to device failure and immune response (*13*).

The quality of recording, capacity to stimulate, and associated lifetime of a device can be boiled down to the device’s ability to resist or overcome increases in electrical impedance. Although the invasive nature of implantable devices causes both acute and chronic tissue damage and remodeling, a focus is placed on the effects on electrical interface quality and lifetime rather than the influence on neurological function. The areas of most intense research efforts center around three main pathways that lead to high impedance: (i) device/electrode degradation due to operation in biological environment; (ii) acute and chronic neuroinflammatory response electrically isolating a probe from neural tissue and causing loss of neurons near the electrode site; and (iii) poor inherent electrode performance.

Not surprisingly, certain approaches to alleviate these issues target multiple aspects simultaneously. For example, improved electrode coatings can help overcome the electrical effects from scar formation, and reduction in device degradation can minimize the activated neuroinflammatory pathways.

#### Degradation

Deterioration of performance can sometimes be linked to deterioration of the physical device. This includes direct mechanical damage of the probe or electrode components (possibly due to insertion), destruction of barrier properties of the passivation layer, or mechanical damage/corrosion of the electrode material (*23*, *24*).

Cracking and delamination can be caused by poor adhesion, defects, and/or unintended mechanical stresses (*13*). Damage to insulating layers is most common in this case and may lead to exposure of metallic interconnects. This has the unintended consequence of introducing parasitic current pathways between tissue and recording system or cross-talk between recording sites. Ingress of water, small molecules, or gasses can have a similar effect, hastening delamination. Dissolution of component materials—an aspect that is used by some to achieve controlled dissolution of devices (*25*)—when unintended can lead to exposure of interconnects to the biological milieu.

Corrosion, or otherwise degradation of the electrode material, leads to a twofold negative effect: destroying the conductive properties of the electrode or interconnect (increasing *R*_s_ and/or decreasing *C*_e_) and possibly releasing toxic by-products into the tissue (increasing the immune response or cell death). Some metals (tungsten and stainless steel, for example) readily corrode in ionic media and/or decompose upon prolonged biasing (*26*, *27*), which has led to the use of other metals, alloys, and organic conductors (as described in the “Improving electrode performance” section). In addition, some organic electrode coatings, such as the CP polypyrrole (PPy), can overoxidize easily due to defective polymer backbone coupling (*28*). Both chemical or electrochemical stability and adhesion issues might be addressed through careful materials selection and/or synthesis.

#### Neuroinflammatory response

The neuroinflammatory response is the response of the immune system within the central nervous system and is composed of a combination of chemical and cellular pathways that come together to metabolize or isolate a foreign body, such as an implanted device. Immediately following implantation, activated microglia attach to the surface of the device and release proinflammatory factors. Shortly thereafter, a dense astrocyte encapsulation envelops the probe, forming a scar (astrogliosis) (Fig. 1B) (*13*). The acute response is initiated to induce wound closure and healing, including recruitment of the inflammatory cells to the injury site. The chronic response [reviewed in previous works (*13*, *29*, *30*)] can be caused by a number of factors but is ultimately implicated in neuronal loss and scar formation, as confirmed by Biran and coworkers (*31*), who compared the chronic response of implanted microelectrodes to acute “stab wounds” using the same microelectrodes. From an electrical interfacing perspective, the repercussions of the astroglial scar and the death or migration of neural cells are (i) the introduction of additional impedance due to the scar/biofilm formation and (ii) the reduction of the magnitude of the input voltage during recordings because living neurons are fewer and farther away (see Fig. 1B).

Because the causes and exacerbations of the neuroinflammatory response can be numerous, so must be the approaches taken to minimize them. Mechanical mismatch between brain and probe and micromotions are both implicated in scar formation. Other factors include recruitment of (and persistence of) bound and soluble inflammatory factors. Hence, the general approaches targeted to combat the immune response have been to modify probe materials and/or form factor to more closely match tissue mechanics and to target coatings that will combat inflammation or “trick” the immune system.

**Fig. 2:**
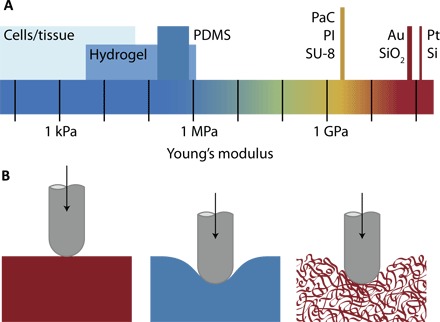
Mechanical mismatch between common probe materials and soft neural tissue. (**A**) Young’s modulus of tissue and common materials discussed. (**B**) Schematic illustrating the mechanical compliance of stiff inorganic materials (Si, metals, oxides; left), compared to elastomers like poly(dimethylsiloxane) (PDMS; middle); by minimizing the critical dimensions, high modulus and nominally rigid materials can be made compliant (right).

Better matching the mechanical properties of the probe with that of the neural tissue is thought to allow the probe to follow the motions of the brain, even if the probe is tethered to a relatively fixed point like the skull (*13*). This mechanical matching is approached from two directions. The first is to make the probe out of polymers that are softer than bulk Si and metals [typically Parylene C (PaC), polyimide (PI), or SU-8]. While an improvement, these materials are still more than four orders of magnitude stiffer than tissue (Fig. 2A). Moving toward more compliant materials—for example, elastomers or hydrogel coatings—helps close this gap. The extent to which bulk material mechanical property matching helps minimize the immune response is not fully understood; recent work suggests that it is the device-scale mechanics that are most important (*13*, *32*). The second approach suggests that stiffer component materials (polymers, metals, and semiconductors) can be used as long as the characteristic dimensions are small enough (1 to 10 μm; subcellular scale) to allow for mechanical compliance (Fig. 2B). The same materials with different cross sections tested in vivo were found to illicit a reduced inflammatory response when the adjoining struts were minimized to the cellular scale—a finding attributed to differences in mechanical properties (*33*). An added benefit is the associated reduction in surface area and, thus, the number of inflammatory cells and proinflammatory soluble factors at the biotic/abiotic interface (*34*). The evolution of form factor for implanted devices has followed these principles and is covered in the “Novel form factors” section.

The implementation of certain passive coatings on implanted devices can diminish inflammatory cascades. In addition to helping mechanical matching, hydrogels or other soft coatings can act as passive sinks that help effectively “clear” soluble inflammatory factors (*13*, *35*). The freedom to functionalize or physically entrap biomolecules on or within surface coatings enables one to regulate cell or protein attachment. Passive polyethylene glycol (PEG) coatings can, for example, minimize adhesion of noncellular proteins that degrade electrode impedance (*36*). Furthermore, passive adsorption or covalent attachment of extracellular matrix (ECM) components can promote neuronal attachment. For example, complex ECM coatings such as fixed astrocyte ECM can effectively reduce microglial activation and can do so more effectively than individual ECM components such as laminin or fibronectin (*37*).

#### Improving electrode performance

As electrode size is reduced to address individual or small populations of neurons, the impedance of the electrode increases. The electrode area-impedance trade-off is well understood (*11*); larger areas reduce *R*_spread_ and increase *C*_e_ while averaging over a larger population of neurons. Although this outcome works well when targeting population-generated low-frequency LFP activity, the need to measure action potentials of individual neurons with a high SNR has led to an overwhelming focus on maximizing *C*_e_ while keeping a small geometric electrode footprint. Similarly, the desire to electrically stimulate small populations of cells with microelectrodes requires high charge injection limits, necessitating low electrode impedance (avoiding large voltage drops at the electrode interface). To this end, electrode coatings and nanostructuring provide a higher effective surface area, the limit of which is soft active materials, such as CPs, that allow for facile penetration of ions at the molecular scale (Fig. 1C).

Flat electrodes, typically exposed metal/alloy films, such as those based on TiN, Pt, PtIr, stainless steel, and IrO_*x*_, have been evolved toward porous and nanostructured variants (*11*) by modification of deposition processes or performing postprocessing steps such as electrodeposition or etching. The nature of interfacial charge transfer can be purely capacitive (as in TiN and stainless steel) or faradic (as in IrO_*x*_)—the latter being acceptable only in cases of confined and reversible redox processes. Nanostructured and porous IrO_*x*_, TiN, and Pt-black allow for a substantial increase in the surface area of the electrode/electrolyte interface and are commonly used for multielectrode arrays. A similar approach has been taken with carbon nanostructures [carbon nanotubes (CNTs) and graphene] (*38*, *39*), as well as their composites, with similar results (*40*). As the microstructural tortuosity is increased and the physical pore sizes are reduced, regions of the film experience higher ionic and electronic resistances, which complicate the equivalent circuit in Fig. 1A, often requiring transmission line models such as that of Bisquert *et al*. (*41*) to describe their impedance.

CPs, in particular, offer a unique advantage for improving electrode performance. Materials such as PPy and poly(3,4-ethylenedioxythiophene) (PEDOT) are most commonly used because of their versatility of deposition/patterning, hygroscopic nature, and resulting excellent mixed conduction properties. They can be patterned through electrochemical polymerization on prepatterned electrodes, vapor phase polymerization, or solution casting and may be readily combined with dispersed nanowires/CNTs (*42*) or graphene oxide (*43*) to boost electrical conductivity. Weak intermolecular bonding and the existence of excess polyelectrolyte such as poly(styrene sulfonate) (PSS) in some CPs (that is, PEDOT:PSS) allow for swelling >100% and, thus, high ionic mobility (*44*) and soft mechanical properties. Hence, ions readily penetrate the bulk of the CP, yielding high volumetric capacitance (*45*). The combined ease of ionic penetration and sufficient pathways for electronic transport yield capacitance per unit geometric surface area more than two orders of magnitude higher than flat metallic electrodes and, thus, improve SNR and increase the capacity for stimulation (*14*, *46*, *47*). For sufficiently hydrated, high ionic mobility CPs, the enhanced mixed conduction properties allow for the *R*_spread_(*R*_e_‖*C*_e_) equivalent circuit model in Fig. 1A to be recovered, where *C*_e_ now represents the volumetric, molecular-level electrical double layer (EDL), rather than a geometric, areal EDL (*48*).

CPs can take on nanotube morphologies (*49*) and can be polymerized through and on hydrogels (*50*) or around cells/tissue (*51*). Furthermore, they can be readily functionalized via physical entrapment and covalent cross-linking with biomolecules and cells, which can effectively blur the biotic/abiotic interface and promote tissue incorporation [covered extensively in previous studies (*13*, *15*, *52*, *53*)]. Many of these functionalization schemes are similar to the passive coatings described above (see the “Neuroinflammatory response” section).

In describing the number of ways that electrical recording can be improved through materials and device engineering, it is clear that no one route is a panacea; the greatest gains come from using multifaceted approaches that target many of the approaches described above. As an illustrative example, Kozai *et al*. (*54*) demonstrate a composite microelectrode that shows stable unit recordings over 5 weeks and significantly reduced neuroinflammation response as compared with Si-based probes. The fiber is based on a 7-μm-diameter carbon microfiber coated with a bioactive functionalization that allows for the probe to readily follow tissue movement (minimizing micromotions) and to effectively prevent ongoing neuroinflammation. The small electrode tip is also coated with PEDOT to provide a low impedance and enhance the SNR of the microelectrode.

### Novel form factors

The escape from rigid, needle-like form factors is bolstered by the inclusion of soft, polymeric, and adaptive passive materials as well as new, ultrathin, and unique form factors. The shift away from microwires and photolithographically patterned or micromachined silicon shanks is desired to address the micromotions and immune response discussed in the “Neuroinflammatory response” section.

The interaction of an implanted device with neural tissue ultimately depends on device-level mechanics (minimizing motion and insertion trauma), not necessarily the bulk mechanics of the component materials. To this end, a device can be made of a low-modulus elastomer, providing a lower mechanical mismatch with biological tissue than, for example, a metallic wire or silicon shank. However, high-modulus materials can be designed with micrometer-scale features to achieve stiffness comparable to their thicker, lower-moduli counterparts (Fig. 2). This device architecture approach (rather than materials approach) is analogous to the compliant and conformable nature of steel wool as compared to bulk steel. These architectures can be composed of micro- and nanoscale wires or fibers, ribbons, and thin substrates or membranes (<10 μm) and arise due to the cubic scaling of bending stiffness with characteristic dimension (*32*). Tissue integration often calls for stretchability (a low-modulus, elastic response to large strain deformations). This can be achieved with inherently elastic materials or through deterministic, composite designs using serpentine structures, wavy structures on prestressed supports, and with mesh-like architectures, as detailed below.

**Fig. 3:**
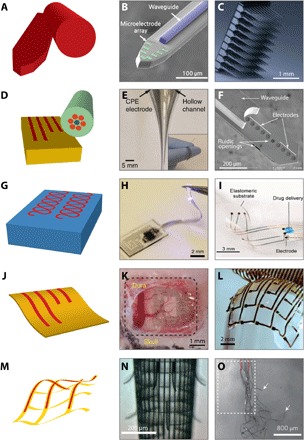
Evolution of form factors for neural interfacing. (**A** to **C**) Rigid, Si-based probes are commercially available and considered state of the art. (B) Si-based Michigan probe, modified with a patterned waveguide. Reproduced with permission from Son *et al*. (*224*). (C) Utah array (Blackrock Microsystems LLC). (**D** to **F**) Thick fiber and polymer-based probes. (E) Thermal drawing of macroscale preforms allows for multifunctional fibers that can bend and flex. A single fiber can contain electrical recording sites (CPE or Sn), guide light, or pass fluid. Reproduced with permission from Canales *et al*. (*55*). (F) Polymer probes (based on PI and SU-8) can also be assembled to support optical and fluidic stimulation and electrical interfacing. Reproduced with permission from Rubehn *et al*. (*56*). (**G** to **I**) Elastomeric probes are generally thick but are compliant and stretchable. (H) PDMS probe with off-the-shelf components and serpentine metallic structures. Reproduced with permission from Park *et al*. (*59*). (I) EDura: PDMS-based probe with electrodes and a microfluidic. Reproduced with permission from Minev *et al*. (*58*). (**J** to **L**) Ultrathin arrays and probes. (K) Neurogrid array: PEDOT:PSS-coated Au electrode sites on 4-μm PaC. Reproduced with permission from Khodagholy *et al*. (*67*). (L) SU-8 and Au array on silk fibroin that can be dissolved away to leave a mesh. Reproduced with permission from Kim *et al*. (*68*). (**M** to **O**) Freestanding mesh probes. (N) Stressed struts allow for global scrolling to form a probe-like geometry or (O) meshes that can be injected through a syringe. Reproduced with permission from Liu *et al*. (*74*) and Xie *et al*. (*75*). The colors used in the schematics on the left correspond roughly to the Young’s modulus scale in Fig. 2A.

The gains achieved by tailoring devices made from intrinsically soft materials or by using geometrical scaling to achieve lower effective stiffnesses can be observed in the evolution of device geometry (Fig. 3). In the engineering front focused on developing new tools, rigid shanks and microwires/needles (Fig. 3, A to C) have given way to elastomeric, ultraconformable, mesh-like, and particle-based probes and stimulators. Note that the use of rigid, micromachined tools, such as Utah arrays and Michigan probes, remains strong in neuroscience due largely to their technological maturity (yield, reliability, and support) and potential customizability through commercial entities rather than through academic laboratories.

#### Fibers and thick polymer probes

Fibers are a robust platform for neural interfacing, often in the form of optical fibers used to pipe light from an external source into a specific region of the brain, for example, for optogenetic applications (see the “Optogenetics” section). Canales *et al*. (*55*) demonstrated that thermal drawing of carefully selected materials can result in multimodal probes for interfacing with the brain and spinal cord of freely moving rodents. Rather than the typical glasses used in fiber optics, the authors used plastics commonly employed for medical devices, in addition to Sn and conductive polyethylene composites (CPEs), to achieve 70- to 700-μm-diameter multielectrode and multimodal probes (Fig. 3, E and F). Ultimately, the probes showed less foreign body response compared to microwires and reduced chronic astrocytic and microglial response, allowing for stable brain machine interfacing for 2 months. Although fibers can be readily patterned from prefabricated macroscale preforms and can allow for meters of nearly identical probes to be simultaneously fabricated, the recording and stimulation sites are currently limited to the tips of the fibers, and input/output (I/O) wiring can be tedious. Alternatively, photolithographic patterning and micromachining can be extended to polymeric materials (for example, PaC, SU-8, and PI) to form thick and rigid probes akin to the Michigan array. For example, microelectromechanical system (MEMS)–based fabrication can allow for 85- to 250-μm-thick multimodal optical, fluidic as electrical-based composite polymer probes (*56*).

#### Low–bulk modulus probes

Elastomeric materials bring to the neural interface the possibility to make probes that are not only flexible but also significantly softer and able to withstand local stretching (as is required for regions such as the spine). An elegant approach is to replace well-known single-site microwires composed of insulated metals with a soft, conductive composite. For example, a PEDOT-based elastomeric composite can be extruded and later insulated to achieve microwires that are five orders of magnitude lower in the Young’s modulus than their tungsten counterparts (*57*). The mechanical properties of some elastomers, such as PDMS, have drawn comparisons with the properties of the dura mater. Minev and coworkers, for example, developed an elastomeric probe they termed EDura (Fig. 3I), which allows for electrical recording and chemical stimulation (*58*). The electrical components are composed of microcracked Au interconnects and Pt-silicone composite electrodes that can accommodate the demanding strains of operating within the spine of a freely moving rodent. EDura can thus record cortical and spinal activity, as well as restore locomotion after spinal cord injury. An alternative approach to allow for stretchable PDMS-based electronic probes is the use of embedded metallic serpentine structures demonstrated by Park *et al*. (*59*). These examples suggest that the elastomeric materials can be incorporated as both passive and active components to impart stretchability and mechanical compliance, allowing for reduced immune response and thus longer implantation lifetime.

#### Ultrathin and hybrid form factors

Ultrathin form factors can be achieved with more rigid polymeric substrates at thicknesses of 1 to 10 μm. Although these materials may have higher moduli (GPa) compared to the elastomers described above (~MPa), their thickness is 10 to 100 times lower, allowing for a lower bending stiffness. Thin PI, polyethylene terephthalate, and parylene films have been used for a number of applications. Epidermal, skin-based probes, for example, are a successful case study in conformal, compliant probes enabling new functionality. These applications use ultrathin “imperceptible” form factors or freestanding serpentine structures to allow for a variety of sensing and stimulation modalities (*60*–*62*). For neural interfacing, thin form factors most readily apply to cases where the probe must lie on a surface without penetrating bulk tissue, as is the case for subdural or epidural two-dimensional (2D) arrays [electrocorticography (ECoG)], although guided insertion of ultrathin penetrating probes has been demonstrated (*63*–*65*). Khodagholy and colleagues have demonstrated 4-μm-thick PaC-based probes, using gold interconnects, and CP (PEDOT:PSS) sensing nodes for both active (*66*) and passive (*67*) ECoG arrays (Fig. 3K). In its most recent iteration, the Neurogrid probe includes 256 electrodes, capable of recording action potentials from the surface of the brain, and has been validated in human patients intraoperatively (*67*). Kim *et al*. showed that electrode grids can be made conformal for ECoG recordings when the PI substrate is thinned down to 2.5 μm (*68*). By patterning the array into a mesh-like structure, and using dissolvable silk fibroin films as a transient carrier support (Fig. 3l), adhesion forces due to water capillarity are enhanced, enabling conformal contact on a cat’s cortex and improving the recording of sleep spindles.

Integration of hybrid components, namely, inorganic semiconductors, enables a degree of higher-level functionality, ranging from logic (amplification and active addressing) to on-board optoelectronic sensing/stimulation, as discussed in the “Optical recording and stimulation” section. Enabled by the ability to grow/pattern high quality semiconductor nanomembranes, researchers have been able to bring high-performance semiconductors to flexible neural interfaces. Viventi *et al*. (*69*) used Si nanomembranes to amplify and multiplex an array of 390 sensing electrodes. The strain induced on the inorganic components was minimized by embedding them at the neutral mechanical plane of the 25-μm-thick array (*69*). Subsequent hybrid probes with inorganic nanomembranes have achieved thicknesses of 8 μm and show reduced lesioning, neuronal loss, and immunoreactivity (*63*).

**Fig. 4:**
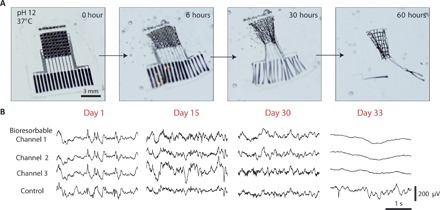
Transient, bioresorbable electronics. (**A**) Transient bioelectronic ECoG array micrographs of active ECoG array with Si transistors under accelerated (high pH) testing conditions. (**B**) Recording from three channels and a control (nonbioresorbable channel) over 33 days in vivo. The transient array is fully functional for >30 days in vivo. Reproduced with permission from Yu *et al*. (*72*).

Some applications benefit from the physical probe completely disappearing after a preprogrammed amount of time. So-called transient electronics have gained interest because the choice of materials and materials thickness can lead to complete dissolution or metabolysis within a preprogrammed time frame. The range of materials includes various conductors, semiconductors, and insulators, with initial biocompatibility and toxicity studies yielding promising results (*70*). One application space that has been targeted is the monitoring of intracranial pressure and temperature for the treatment of traumatic brain injury (*71*). Mapping or localization of epileptic networks may also find utility in transient ECoG monitoring, where follow-up surgeries are commonplace to remove the devices after weeks or where ambulatory intracranial recording may be desirable for months. Yu *et al*. (*72*) have demonstrated these hybrid, transient ECoG arrays. Both passive and active matrix-addressed probes were fabricated on 30-μm poly(lactic-*co*-glycolic acid), with individual materials used dissolving within 1 to 6 months (Fig. 4).

#### 3D mesh-based form factors

The most unconventional probe geometries aim to minimize the volume of the probe to develop an interface that blends into the surrounding tissue. One approach is to embed interconnects and electronics in a mesh-like structure, around which cell and tissue can intercalate (*73*). Similar to the geometry of the mesh ECoG probe described above (*68*), 3D macroporous, nanoelectronic probes have been developed for neural interfacing (Fig. 3, N and O) (*74*, *75*). The probes comprised 1-μm-thick elements with SU-8 as a strut/support material, as well as metal interconnects, and sensing elements including nanowire transistors and Pt electrodes. Liu *et al*. (*74*) showed that the mesh structure can be engineered with respect to the dimensions and mesh cell geometry to tailor the transverse and lateral bending stiffness to allow the structure to be injected from a syringe. Although such a probe shows promise, especially injection through low profile (100-μm-diameter needles), the I/O connectivity must be performed after injection. An alternative mesh structure, demonstrated by Xie *et al*. (*75*), resembles a traditional probe-like structure (with standard I/O connectivity), where built-in strains control the local geometry, allowing for a global scrolling of the probe into a tube-like mesh. The probe recorded LFP and unit activity from a rodent somatosensory cortex. Notably, the ~100-μm-diameter acute void left after insertion was able to “backfill” with neurons. Despite a higher astrocytic response, and lower neural density in the probe core, neuronal processes readily grow among the mesh electronics (*75*). Ultimately, mesh-based probes feature unique biocompatibility, which has been attributed to the micrometer-scale features, open/macroporous structure, and the resulting low bending stiffness quoted as four to six orders of magnitude smaller than previously reported neural probes composed of Si, carbon fiber, and PI with thin-film electronics (*75*). These probes are promising as an approach that minimizes chronic immune response but require unconventional insertion protocols. In addition, they are limited by the lack of relative or global control of precise sensor placement stemming from the compliant mesh and the evolving positioning due to tissue rearrangement and built-in strains.

#### Particle form factors

One of the most creative approaches to reduce the active components of implanted probes is to do away with interconnects altogether. Micro- and nanoparticles can be considered the simplest implementation of an external “probe” used for specific or localized stimulation or recording and, with other modalities described below (see the “Optical stimulation,” “Magnetic stimulation,” and “Ultrasound stimulation” sections), require untethered “wireless” operation. One example is the concept of “neural dust”: cellular-scale particles recently put forth by Seo and coworkers (*76*, *77*) that rely on individual, freestanding sensing nodes ~10 to 100 μm in size and scattered through the brain target area. The neural signal is recorded as a differential signal between two electrodes on the node, which electromechanically modulates a piezoelectric crystal. The piezoelectric modulation varies the ultrasonic backscatter that is interrogated and recorded by a transceiver device implanted subdurally. This concept is still in its infancy. Although a large-scale demonstrator (800 μm) has been reported (*77*), challenges include downscaling node size while minimizing SNR losses, addressing power considerations of subcranial ultrasonic transceivers and implantation strategies.

#### Implantation strategies

As probe geometries deviate from heavily used commercial probes based on commonly used geometries, the ability to handle, implant, or control the placement of devices becomes nontrivial. Of the technologies described herein, a number of general strategies can be described. Transient or removable shuttle materials are commonly used to allow for handling and insertion. For example, epoxy or SU-8 removable shanks or microneedles can be used to guide insertion before removal (*63*–*65*). Alternatively, the support material can be dissolved, for example, materials such as silk, sugars, PEG, poly(lactic acid), or gelatin (*13*). Mechanoadaptive approaches present an alternative route, potentially removing the need for additional material that displaces/destroys tissue. Such an approach relies on a change in modulus upon insertion. The mesh-based probe shown in Fig. 3N was frozen in liquid nitrogen before insertion (*75*)—a debatable approach likely to cause thermal shock of surrounding tissue. Alternatively, Capadona and colleagues (*78*) have developed mechanically adaptive bioinspired nanocomposites that change mechanical properties on exposure to physiological conditions. The material’s Young’s modulus changes from 3.4 GPa to 20 MPa on insertion, allowing for reduced neuroinflammatory response. A similar result has been achieved by Ware *et al*. (*79*) with ternary thiol-ene/acrylate polymer networks, which were used as probe substrates with patterned electrodes and showed minimal water uptake.

The most nonconventional geometries are the most challenging to implant. The injectable mesh electronics are delivered through a syringe (*74*), with reduced control of placement once they exit the syringe tip. Particle-based probes are perhaps the most challenging; the question remains not only how to place them but also will they stay there and for how long before diffusing away or being metabolized?

### Intracellular recording

Although extracellular recording and stimulation have been heavily investigated, especially due to its immediate relevance for clinical neuroscience, intracellular recording can enable high SNR recording of individual cells (without traditional patch-clamp approaches) using nanostructures such as nanowires (*80*–*82*), mushrooms (*83*), and straws (*84*). In many cases, it is electroporation that allows for recording of transmembrane potentials; however, the use of carefully functionalized wires and nanostraws, patterned with a band of peptides or hydrophobic organic molecules, allows a probe to penetrate through the lipid bilayer for true intracellular access (*85*). These approaches push the limits of neural interfacing and nanotechnology and, in the process, allow for direct measurements of the variation in transmembrane potential and potentially fluidic access to the cytosol. However, significant challenges to implementation exist, including the placement, micromotion, and wiring of individual nanowires to external recording/stimulation systems (*86*, *87*).

### Active interfacing

To maintain a high recording quality, ideal signal processing would call for signal amplification as close to the recording site as possible. Using silicon complementary metal-oxide-semiconductor (CMOS) technology, this can be readily achieved and has been implemented in in vitro multielectrode arrays (MEAs) (*11*). With limited space constraints, it can be challenging to tightly integrate multiple transistors into a recording array meant for implantation/tissue integration. For this reason, active recording sites have been targeted, whereby the passive electrode is replaced by a transistor. Transistors have an inherent amplification or gain, whereby a small variation in the effective gate voltage, in this case, the effective potential due to tissue activity, leads to a large change in the current through the transistor channel. Hence, early work by Fromherz and colleagues (*88*, *89*) demonstrated transistor arrays that sensitively transduce neural firing events. Depending on the materials or transistor type, either the gate dielectric or the transistor channel itself is placed in direct contact with the biological environment. A recent example garnering attention for implantable applications is the organic electrochemical transistor, where the channel material is a CP (*90*). In this case, the high volumetric capacitance of the material (such as CPs discussed in the “Improving electrode performance” section) yields high currents and high effective gains, which allows for improved SNR recordings of physiological and pathological activity (*66*, *91*), as well as stimulation (*64*). Transistors have also taken on nanoscale form factors to allow for in-tissue (*73*) or even intracellular integration (*80*) to record activity and to decode neural circuitry (*92*).

One area that should not be overlooked, yet is not covered in detail here, is the higher-level electronics required for multimodal recording and stimulation systems to move beyond the laboratory. This includes active matrix and multiplexing capabilities to increase recording density and minimize the number of physical wires requiring external connection. These active approaches have been used to realize high-density ECoG arrays (*69*) and implantable CMOS-based Si probes with electronic depth control (*93*, *94*). To minimize the external electronics and improve recording quality, higher-level logic should be integrated into implantable devices, including amplifiers, spike detection and closed-loop capabilities, calibration, and other analog and digital circuitry (*11*, *93*).

### Electrical-based physical and biochemical sensors and stimulators

The library of electrical-based sensors and actuators that can be integrated into implantable devices is extraordinary, and their role in modern neuroscience tools is unquestionable. The modalities include sensitive pressure and temperature sensing for monitoring tissue state, wound healing and/or blood flow (*95*, *96*), and a variety of chemical sensors. Most of the electrical-based chemical sensors rely on electrochemical reactions or capacitive changes due to specific binding events. In either case, specificity is facilitated by a detector unit such as an ionophore (for ion detection) or enzymes, which lead to direct or indirect charge transfer or variation in local charge that is transduced as electrical signals. The literature on specific binding for biosensors is vast (*94*). Alternative approaches call for the detection of the electrochemical signature of a molecule (that is, from cyclic voltammetry) to detect its presence. In this regard, fast-scan cyclic voltammetry (FSCV) can be beneficial but is normally hindered by environmental noise. Coupling FSCV with an electrochemical transistor may overcome these hurdles and has been demonstrated for measuring micromolar dopamine concentrations (*97*).

Electrical control of chemical stimulation presents a favorable advantage over fluidic approaches. Fluidic delivery can lead to deleterious solvent effects and increases in physical and osmotic pressure in the tissue. One route to deliver drugs or biomolecules in an implanted form factor, upon demand, has been to electrochemically release entrapped molecules loaded into CPs (*98*). This has the downside that the active eluting electrode has a very limited capacity for the biomolecule of interest, limiting delivery lifetime. Organic electronic ion pumps (OEIPs) allow for electrophoretic delivery of charged biomolecules (such as neurotransmitters). This means that no fluid is delivered at the release site. Hence, OEIPs have been used to affect sensory function in a guinea pig cochlea (*99*), to affect pain pathways in the spinal cord (*100*), and, while not implanted, to affect pathological epileptiform or hyperexcited neural activity in rat brain slices in vitro (*101*). Because fluidic transport is not required, they can be patterned using common photolithographic techniques on flexible substrates at small sizes. OEIPs require a reservoir of solubilized ions and are often limited by the capacity of their driving source and target electrodes; however, the ability to continuously regenerate the electrodes has been proposed (*102*).

Because the transmembrane potential governs the activity of neurons, electrical (and biomolecular) interfacing has been the most tenured approach to recording or stimulating neurons. Although the electrode is still a mainstay in much of neuroscience research, diagnostics, and therapy, in many cases, it cannot always provide the SNR recording, stimulation, and recording specificity/reliability required to answer many questions or solve certain problems. For this reason, significant efforts focus on improving or finding new modalities for interfacing that achieve unprecedented specificity, localization, and noninvasiveness, among others. By bringing together synthetic chemistry, molecular biology, genetics, and cellular biology, as well as electromagnetic radiation in various forms, new tools for stimulation and recording based on engineered probes, particles, molecules, and proteins can be realized.

## OPTICAL RECORDING AND STIMULATION

In the experimental setting, the advantages of using visible and infrared (IR) light as an input control source or readout signal for neural activity are multiple-fold including scalable intensity to allow for analog signals, penetration into tissue (dependent on wavelength), and safety (more so than the ultraviolet component of the spectrum). These same advantages translate to the clinical realm, where less-invasive modalities often allow access to a larger pool of candidate patients, and safety is paramount. The trade-off between commonly used noninvasive imaging [such as x-ray, computed tomography, and magnetic resonance imaging (MRI)] and control modalities (magnetic stimulation and ultrasound), compared to the ones described below, is depth for resolution: Visible- and IR-based techniques are still limited by the inherent scattering of these wavelengths by lipid-rich brain tissue but, within their useful working distances, are able to resolve single-neuron and subcellular information. Current and future engineering is working to increase the functional depth of signal readout based on tool design, largely by moving their spectra further into the IR, which is less affected by scattering in the brain.

### Optical recording

**Fig. 5:**
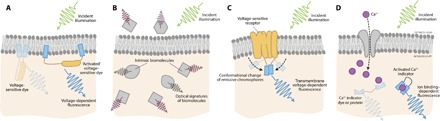
Optical neural recording strategies. Diverse approaches use the neuron activity–dependent modulation of light absorption or emission to monitor single-cell or population activity. (**A**) Voltage-sensitive dyes (for VSDI) accumulate within the membranes of neurons and change their conformation depending on the membrane potential, leading to changes in light emission. (**B**) Intrinsic imaging monitors absorption and emission of wavelengths that correlate to those of metabolic biomolecules whose numbers or composition depends on neural activity. (**C**) GEVIs are engineered proteins that consist of a voltage-dependent domain that is embedded in the neuron membrane and a fluorescent protein. Membrane potential changes the conformation of the protein, thereby changing fluorescence emission. (**D**) GECIs are engineered proteins that have a calcium-binding domain (blue) connecting a fluorescent indicator (loop). Protein conformation changes with calcium binding, leading to a change in fluorescent emission. Both (C) and (D) are genetically encoded.

Light is used both experimentally and clinically to read out patterns of natural or induced neural activity. Voltage-sensitive dye imaging (VSDI; Fig. 5A) relies on small molecules that change their emission profile based on local potentials; commonly used variants are the ANEP and RH families [comparison of different dyes in vivo from the study of Grandy *et al*. (*103*)]. After loading these in a cortical area of interest using a syringe or in a single neuron using patch-clamp techniques, the dye accumulates in the neuron membrane, and a microscope with a photodetector array is used to rapidly measure signals from the dye of a specified “active” wavelength; changes in the intensity at this wavelength are correlated with changes in the local potential and therefore neuron activity. Developed and characterized predominantly between the mid-1980s and 2000s, these showed early advantages of facile delivery into live preparations, including frogs (*104*), rats (*105*), and nonhuman primates (*106*), and repeatability of measurements over hour time scales. Emissive changes are rapid, on the order of milliseconds, but are limited in their use across preparations and tissue environments because of uneven and varied cellular uptake; for this reason, one of the limitations (or advantages) is utility in being able to image large cortical areas (as opposed to single-neuron resolution). Similar to voltage-sensitive dyes, in concept, inorganic quantum dots have also been proposed as voltage sensors because of their superior photo-stability (*107*, *108*).

A parallel and less-invasive optical approach to cortical imaging takes advantage of the intrinsic and characteristic absorption of visible and IR wavelengths by molecules in blood and neurons that vary their optical properties depending on neuron metabolic activity. Light corresponding to the wavelength of a given molecule or signature is introduced and recorded via fiber optic; the recorded signal will vary depending on the metabolic load. In theory, this signal correlates with the overall amount of neural activity. This “intrinsic imaging” (Fig. 5B) was first shown to be useful in mapping ocular dominance columns in cats and nonhuman primates (*109*) using 500- to 800-nm wavelengths, even being able to show orientation columns. This approach requires minimal equipment, as shown by incorporation of near-IR intrinsic imaging during functional mapping of cortical sites of primary and secondary language function in human patients undergoing partial lobectomy for epilepsy (*110*). The use of IR wavelengths has been shown to be able to penetrate the skin and skull and allow for completely noninvasive mapping of cortical motor activity (*111*).

Another approach developed over the last 20 years for fast-scale readout of neuron activity is genetically encoded indicators. These indicators have used multiple molecular engineering approaches to couple proteins that are intrinsically fluorescent with other proteins that undergo conformational changes in response to salient cellular events—commonly, voltage-sensitive proteins that embed in the membrane and change conformation in response to membrane potential [genetically encoded voltage indicators (GEVIs); Fig. 5C] or ones that have calcium-binding domains and have a calcium concentration–dependent conformation [genetically encoded calcium indicators (GECIs); Fig. 5D]. It should be noted that sensors for other ions and small molecules have been described, but GECIs are the most widely used and well developed. The main difference between these two families is the type of signal that is read out by the change in fluorescence: GEVIs are able to relay action potentials as well as subthreshold (non–action potential) changes in membrane potential, whereas GECIs report changes in calcium concentration, which is a direct proxy for action potentials (intracellular calcium in the neuron is tightly regulated to approximately 10,000 times less than extracellular Ca^2+^). The practical trade-off is that GECIs are much more well established for use in vivo than GEVIs, although this is changing. Since initially being reported in the late 1990s (*112*), GEVIs have undergone multiple iterations (*113*–*118*), initially being useful for only rough estimates of *Xenopus* oocyte membrane potential over a limited range of voltages, but are now able to track single action potentials and subthreshold potentials at physiologic speeds in neurons. This roughly tracks the progression of GECI development, which was initially useful in a limited number of contexts (*119*, *120*), but, through multiple rounds of rational and screening-based modifications (*121*, *122*), GCaMP, the archetypical member of this protein class, is now useful for the simultaneous readout of thousands of neurons (*123*–*125*). Because both classes of optical readout tools are genetically encoded, they may also be used in conjunction with a multitude of standard molecular tools in experimental neuroscience that allow for their selective introduction into neurons defined by their anatomic region, connectivity patterns, genetic markers, or a combination thereof.

Optical measurements of neural activity allow for the theoretical online readout of membrane potential across many neurons simultaneously. Genetically encoded sensors, voltage-sensitive dyes, and intrinsic imaging have each taken a different approach to imaging neural activity. Of the three, only intrinsic imaging has found utility in humans. The use of GEVI/GECI and VSDI in humans is unlikely in the near future. Although genetically encoded sensors have been shown to work in multiple mammals and cell types, the use of all genetically encoded tools requires gene delivery, which, in the central nervous system, would almost certainly require the use of a virus (gene therapy) and is not on the horizon for this particular tool set (although trials are under way for other genetically encoded tools; see the “Optogenetics” section). VSDI has been shown to work in stem cell–derived human tissues (*126*); however, characterized voltage-sensitive dyes have not been approved by the U.S. Food and Drug Administration (FDA) [nevertheless, note that an FDA-approved compound has recently been found to have voltage-sensitive optical properties (*127*)]. However, intrinsic imaging has been used both intraoperatively and at the bedside in humans, and the signals have been validated using EEG and functional MRI (fMRI); however, it does not provide signal quality improvements (speed or depth) over either of these modalities and is unlikely to become a clinical mainstay.

### Optical stimulation

**Fig. 6:**
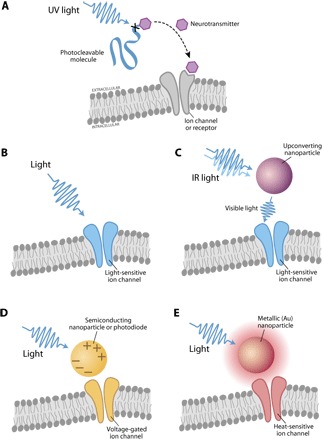
Schematics of optical stimulation methods. (**A**) Two-photon uncaging uses biologically inert neurotransmitters that become available for receptor binding after their caged moiety absorbs photons of the correct wavelength, thereby giving researchers the ability to temporally and anatomically control receptor activation. UV, ultraviolet. (**B**) Optogenetic stimulation with genetically encoded light-sensitive ion channel (for example, channelrhodopsin). (**C**) Upconverting neural stimulation by upconverting nanoparticles acting on light-sensitive ion channels. (**D**) Photoelectric stimulation with semiconducting devices or particles acting on voltage-sensitive ion channels. (**E**) Photothermal stimulation with metallic (for example, gold) nanoparticles acting on cell membrane or heat-sensitive channels.

As a control mechanism, light has revolutionized the neuroscience toolbox, initially as the trigger for experiments using “caged” neurotransmitters, whereby the ligand is inactive because of conformation or linkage with a separate molecule until it absorbs light of the correct wavelength, which effectively unbinds the now active molecule from its photosensitive cage (Fig. 6A). After being uncaged, the active molecule (that is, neurotransmitter) is free to bind and activate any receptors in the vicinity, allowing neuroscientists to precisely examine the effect of selectively (in space and identity) activated receptors [for instance, at the resolution of single spines of the dendrites of a single neuron (*128*)]. One example of a caged neurotransmitter compound is methoxy-nitroindolino (MNI)–linked version of the excitatory neurotransmitter glutamate; MNI-glutamate can be uncaged using light from a 720-nm, two-photon laser setup. Many compounds have been developed for caging molecules with various properties; most of these are excited by 300- to 400-nm wavelengths (allowing use with a 700- to 800-nm, two-photon system) (*129*). Obvious limitations to this approach are the limited number of characterized compounds that may be caged, the need to know a priori where specific receptors are precisely located, and the need to deliver a caged compound, which may be challenging for most in vivo experiments aimed at linking stereotyped patterns of neural circuit activity with behavior.

#### Optogenetics

Beyond uncaging, a separate avenue to control neurons with light is the adaptation of microbial opsin genes for neuroscience (*130*). These “optogenetic” tools encode ion channels or pumps that are in a closed or inactive state until absorbing photons of the correct wavelength (Fig. 6B); they then either open (in the case of channels, such as the cation channel ChR2) or move a step through their ion pumping cycle (in the case of pumps, such as the chloride pump NpHR). Their ion selectivity allows for the depolarization (activation) or hyperpolarization (silencing) of preselected populations of neurons, ranging from single neurons in culture to awake, behaving animals and from worms to nonhuman primates (*131*, *132*), and with a live human trial under way. Current work in engineering these tools has centered on shifting the activation spectra further into the red (*133*–*135*) to decrease light scattering and improve penetration depth [calculator available at www.optogenetics.org/calc (*136*)] and on creating or discovering variants that conduct chloride (*137*–*140*), with ongoing work to create potassium-selective tools (*141*). More exotic approaches, including the use of lanthanide-doped upconverting nanoparticles to convert incident IR light to shorter-wavelength visible light (capable of activating current opsin proteins), have shown early promise in moving toward stimulation wavelengths that penetrate deeper into the tissue (Fig. 6C) (*142*–*144*). This approach is hindered by the low nanoparticle quantum yield and therefore requires incident power that is several orders of magnitude higher than standard optogenetic stimulation. With an improved quantum yield, this approach may provide a separate avenue to avoid scattering limitations in optogenetic experimental design and therapeutic application.

At a mechanistic level, the opsin proteins are covalently bound to all-trans retinal, which acts as the light-sensing moiety. There is a sufficient amount of native retinal in the mammalian brain that no separate cofactor needs to be introduced in order for optogenetic tools to function. Unlike the small-molecule caged neurotransmitters, optogenetic tools, as implied by their name, are genetically encoded proteins and are typically delivered to their neural targets as a viral payload. Although gene therapy has been slow for many translational opportunities, the ability to selectively turn specific neurons on or off with light may be useful in many psychiatric and neurological diseases. Among numerous insights and approaches to understanding and curing disease of the nervous system using optogenetics, these tools have been shown to restore some aspects of light detection in mice (*145*, *146*) blinded but with circuitry of the retina largely intact. Following the results in restoring light-sensing function with human retinal explants (*145*), a pilot clinical trial is being conducted for gene therapy with optogenetic tools to treat retinitis pigmentosa (identifier NCT02556736), the results of which may inform and direct future avenues of human intervention with optogenetics. However, the main value of optogenetics has always been for basic science discovery itself.

#### Photoelectric neural stimulation

Another pathway for optically enabled stimulation of neural activity is achieved by exploiting the photoelectric effect. Both bulk semiconducting films and semiconducting nanoparticles have been used to this effect (Fig. 6D) (*147*–*153*). The principle is similar to the operating mechanism of photodetectors or solar cells, where light is absorbed, generating electron/hole pairs, which can be redistributed/collected to affect the spatial charge distribution. Recently, the Palanker group has used an array of high-resolution (pixel size, 70 μm), high-density silicon photodetectors for robust stimulation of neural activity at low irradiance (0.2 to 10 mW mm^−2^) (*154*–*156*). Combined with ease of implantation and wireless stimulation, this approach paves the way for efficient retinal prosthesis in blind patients. Semiconducting nanoparticle stimulation approaches (*149*, *150*), on the other hand, may have an additional benefit of being targeted to stimulate certain neural types (for example, ganglion cells). However, this mode of stimulation still requires development for in vivo applications (*157*).

#### Photothermal neural stimulation

Light can be applied to generate heat, which can be used for modulating neural activity (*158*). For example, short-pulse IR light has been demonstrated for photothermal stimulation as a result of thermally sensitive ion channels or due to a change in cell capacitance (*159*). Recently, it was reported that IR light could inhibit neural activity with a long and weak exposure (*160*). This research is ongoing, and the mechanism of inhibition is poorly understood. Photothermal stimulation may be enhanced further by applying materials that absorb the stimulating light. These materials range from conjugated polymers (*160*, *161*) to gold nanoparticles (*162*–*165*). In particular, gold nanoparticles/nanorods can exhibit very strong light absorption at their plasmon resonance and convert light to heat during the plasmon resonance decay (*166*, *167*). Lower incident power was sufficient for stimulation/inhibition with both gold nanoparticles and conjugated polymers, which is especially desirable for retinal applications (*161*, *162*, *168*). Furthermore, genetically targeted photothermal stimulation could be achieved by expressing a heat-sensitive ion channel such as TRPV1 (Fig. 6E) (*169*). Alternatively, nanoparticles can be functionalized with antibodies against specific ion channels or receptors, avoiding the need for gene therapy (*162*).

### Hardware for optical stimulation and recording

Optogenetics has largely driven optical hardware development for neuroscience over the past decade (*170*). The available light sources for neural stimulation are lasers or light-emitting diodes (LEDs). Lasers have been chosen for most optogenetic experiments due to their high power and efficient coupling with fibers for neural stimulation. More advanced design of multiple fiber systems can achieve optogenetic stimulation of many brain sites (*171*, *172*). LEDs are smaller, less expensive, and available in various wavelengths. However, they sometimes do not provide enough power, and their noncoherent light couples inefficiently with optical fibers. Nevertheless, the smaller size of LEDs makes them advantageous for integration into the working end of implantable devices, where they can be used to directly stimulate tissue or can be coupled to on-device waveguides. Micro-LEDs implanted at both deep brain and peripheral sites can be powered wirelessly and have been shown to be sufficient to control neural activity in awake, behaving rodents through optogenetic manipulation (*59*, *63*, *173*–*175*). Many new devices integrating on-probe waveguides and/or micro-LEDs use the approaches discussed for electrical probes (see the “Novel form factors” section), for example, using elastomeric materials, deterministic serpentine structures, and/or thin form factors to impart mechanical compliance and stretchability. These next-generation implantable optical devices show promise for chronic studies: Histological tests suggest that the flexible micro-LED devices produce much less glial activation and lesions than do traditional optical fibers (*63*).

Imaging hardware continues to advance, with significant efforts devoted to high-fidelity recording (and stimulation) during freely moving animal experiments. The fiber-based approach is advantageous for its straightforward implantation (*176*, *177*); however, fibers can only record the total fluorescence from the populations of neurons within the attenuation depth of the illuminating fiber tip. Fiber bundles and their attachment with gradient refractive index lenses enable imaging capabilities with fiber approaches (*178*, *179*). Recently, it was shown that seven single fibers can be implanted at various locations of a brain, enabling simultaneous recording in seven different regions of the brain in freely behaving rodents (*180*). As an alternative to fiber optics, miniaturized, head-mounted microscopes for direct imaging have been developed (*170*, *181*–*183*) and enable stable imaging of thousands of cells over 1 month (*124*).

## MAGNETIC RECORDING AND STIMULATION

### Magnetic recording/imaging

Magnetic modalities play a major role in both neural recording and stimulation due to their noninvasiveness and high resolution. Most currently used magnetic tools do not require any surgery, implantation, or ingested substances. The most widely used magnetic recording technique is MRI. MRI works by interrogating the magnetic moments (spins) of hydrogen protons that are strongly influenced by their chemical environment. A strong magnet aligns the proton spin in, for example, water molecules; a radio frequency MRI scanner perturbs these spins and then measures their relaxation. Various paramagnetic contrast agents such as gadolinium chelates (*184*–*186*) have been developed to enhance MRI signal. Recently, genetically encoded contrast agents based on metalloproteins (for example, ferritins) have also been developed for long-term cell labeling (*187*, *188*).

fMRI is used to measure neural activity indirectly. In this case, contrast arises from the oxygen carrier protein hemoglobin. Active neurons consume more oxygen, leading to a decreased oxygen concentration in these brain regions. This phenomenon is called blood oxygen level–dependent (BOLD) effect (*189*). The deoxygenated hemoglobin is more paramagnetic than oxygenated hemoglobin, which leads to detectable magnetic contrast. However, biological effects on oxygen concentration and blood flow limit the spatial and temporal resolution to ~1 to 2 s and with a spatial resolution of around 1 mm (*190*). To address these limitations, several other molecular imaging methods have been developed to monitor brain activity (*188*, *191*, *192*). Similar to recording brain activity with fluorescent calcium dyes, Ca^2+^-sensitive domains can be linked to MRI-based contrast agents such that spin relaxation is coupled to changes in Ca^2+^ concentration, yielding response time down to 100 ms (*193*). Alternatively, Atanasijevic *et al*. developed a method to control the aggregation of iron oxide particles upon binding of Ca^2+^ (*194*). Furthermore, manganese ions (Mn^2+^) can be used as a contrast agent to image neural activity due to its high chemical and functional similarity to Ca^2+^ (*195*). Hence, the contrast produced by Mn^2+^ is more directly related to neural activity in comparison to the BOLD method. The main disadvantage of Mn^2+^ is its toxicity at high concentration (*195*). More recently, molecular probes have also been developed to sense neurotransmitters such as dopamine (*196*) and glutamate (*197*), which play an important role in neural signal transduction.

Magnetoencephalography (MEG) directly measures the local magnetic field produced by neural currents and can be used for mapping brain activity in a manner analogous to and with similar temporal resolution as EEG (~1 ms). Because the magnetic fields are less distorted than electrical fields by the skin and skull, MEG has higher spatial resolution (~1 mm) as compared to EEG (~1 cm) (*198*). However, MEG is much more expensive than EEG, requiring highly sensitive magnetometers such as superconducting quantum interference devices and well-isolated rooms to measure the small magnetic fields generated from the neural currents in the brain.

### Magnetic stimulation

Transcranial magnetic stimulation (TMS) has been applied as a noninvasive stimulation method for the understanding of neural activity (*199*, *200*) and for therapy applications (*201*, *202*). In contrast to using uniform field in the case of MRI, TMS applies a fast pulse of magnetic field perpendicular to the coil plane, which induces an electric current at the surface of the brain for neural activation (*203*). TMS has been applied as a possible treatment for Parkinson’s disease, epilepsy, stroke, and pain (*201*, *202*). However, TMS can only stimulate neurons near the outer surface of the brain. The development of micromagnetic stimulation (μMS), using micrometer-scale coils, allows for improved resolution of magnetic stimulation, potentially allowing for implantation (*204*, *205*). Previous studies are motivated by claims that the volume of activated neurons arising from μMS should be larger than that of similarly sized electrical stimulation devices, suggesting a reduction in the role of glial scaring (*204*); however, the study stops short of directly demonstrating μMS through a glial scar. Nevertheless, microcoil devices may provide a route to better understand the mechanisms of electromagnetic neurostimulation and may be a promising alternative to existing technologies. Despite the improvements in magnetic stimulation, neither TMS nor μMS can stimulate specific types of neurons.

**Fig. 7:**
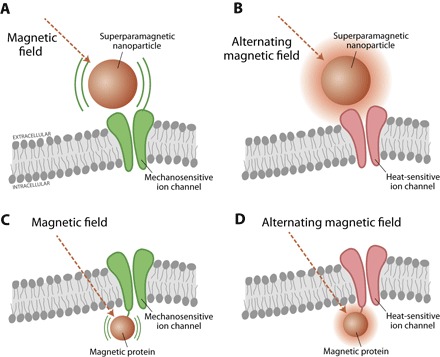
Schematic of suggested cell-specific magnetic stimulation methods. (**A** and **C**) Magnetomechanical stimulation with superparamagnetic nanoparticles or magnetic proteins acting on a mechanosensitive channel (for example, TREK1). (**B** and **D**) Magnetothermal stimulation with superparamagnetic nanoparticles or magnetic proteins acting on a heat-sensitive channel (for example, TRPV1). The magnetic nature of the protein-based stimulation approaches (C and D), that is, coexpression of ferritin with the mechanosensitive/heat-sensitive ion channels, is currently debated and requires further investigation and confirmation of the proposed underlying mechanisms.

Recently, cell-specific magnetic stimulation methods were suggested by applying the combination of magnetic nanoparticles and certain ion channels. One possible mechanism put forward is the activation of mechanosensitive channels (for example, TREK1 channel) by the magnetic force from synthetic magnetic nanoparticles (Fig. 7A) (*206*–*208*). Another mechanism suggested has been the application of radio frequency alternating magnetic fields to the magnetic nanoparticles for the generation of heat, which can be used to activate heat-sensitive channels such as TRPV1 (Fig. 7B) (*209*–*211*). Genetically encoded magnetic proteins such as ferritin can also be coexpressed with TRPV4 or TRPV1 channels at the same time (*210*, *212*), which was suggested to control neural activity and animal behavior in vivo (Fig. 7, C and D) (*212*, *213*). In addition, neural stimulation was reported with a single magnetic protein, MagR, but no explanation was provided as to the mechanism of operation, downstream ion channel coupling, etc. (*214*, *215*). The advantage of these genetic approaches would be that they can achieve genetically targeted neuron stimulation without the implantation of optical devices or injection of nanoparticles. However, a theoretical calculation demonstrated that energy produced from the MagR and ferritin proteins is several orders of magnitude lower than thermal energy in these experiments (*216*). Hence, further effort is required to confirm and explain these results and the underlying mechanisms.

## ULTRASOUND RECORDING AND STIMULATION

### Ultrasound recording

Traditional ultrasound-based recording of activity relies on transduction of sounds (mechanical waves). Ultrasound imaging sends pulsed ultrasonic waves (>20 kHz) into the body and receives the echoes backscattered by tissues or fluids, which absorb incident vibrations differently, thus producing an image. Functional ultrasound (fUS), for example, can be used to measure the cerebral blood flow as a result of neural activation. Compared to fMRI, fUS results in similar spatial and temporal resolution. Efforts in this field focus on increased frame rate and resolution using plane-wave illumination (*217*) and miniaturization of hardware for behavioral studies (*218*). In addition, ultrasound contrast agents based on genetically encoded nanostructures might be applied for molecular imaging with ultrasound (*219*). Finally, as mentioned in the “Novel form factors” section, ultrasound has recently found use as an interrogator in the neural dust recordings of Seo and coworkers (*76*), facilitating the transmission of extracellular potentials transduced through piezoelectric modulated ultrasound backscatter.

### Ultrasound stimulation

Ultrasound stimulation has also attracted increased attention recently because of its high spatial resolution and noninvasiveness. Ultrasound stimulation has been demonstrated in brain slices (*220*), in retina (*221*), in vivo in mice (*222*), and even in humans (*223*). Although higher-frequency ultrasound provides better spatial resolution, lower-frequency ultrasound enables deeper brain penetration and is therefore more effective for brain neuromodulation (*224*, *225*). The mechanism for neural stimulation with ultrasound is still under investigation.

**Fig. 8:**
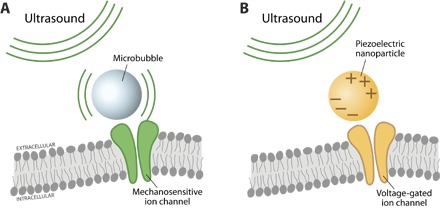
Schematic of suggested ultrasonic stimulation methods. (**A**) Ultrasonic stimulation with microbubbles acting on mechanosensitive channels (that is, TRP-4). (**B**) Ultrasonic stimulation with piezoelectric nanoparticles acting on voltage-sensitive channels.

To enhance contrast and specificity for neural stimulation, either genetically encoded ion channels or nanoparticles can also be used, similar to the techniques covered in Figs. 6 and 7 for optical and magnetic modalities. Recently, it was reported that mechanosensitive TRP-4 channels, together with microbubbles, could sensitize neurons to ultrasound and result in behavior effects in *Caenorhabditis elegans* (Fig. 8A) (*226*). The authors termed it as “sonogenetics” in an analogy to optogenetics. The successful expression and function of TRP-4 in mammalian neurons are still required for its general utility as an ultrasound stimulation tool. Piezoelectric nanomaterials such as barium titanate nanoparticles have also recently been applied for neural stimulation (Fig. 8B) (*227*). This preliminary work demonstrates that these particles could convert ultrasound waves to electric fields and activate voltage-gated ion channels. In a similar vein, core-shell CoFe_2_O_4_-BaTiO_3_ nanoparticles have been reported for magnetoelectric stimulation of neural activity via magnetostrictive-to-piezoelectric coupling (*228*). However, further work is needed to thoroughly characterize the magnetoelectric effects of the nanoparticles and the cellular response from this stimulation method. To further confirm these observations and support the mechanisms, more rigorous statistical studies with both cultured neurons and in in vivo studies are needed.

## ODDS, ENDS, AND THE AMBIGUITY IN CLASSIFICATION

Certain modalities are difficult to categorize in one of the above simple modalities: electrical, optical, magnetic, or mechanical (ultrasound). This includes some common imaging techniques and their derivatives, such as positron emission tomography (PET) and single-proton emission computerized tomography, which are also alternative imaging techniques to MRI for functional whole-brain imaging. These techniques require the injection of radioisotope (tracers) into patients and the detection of gamma radiation from the tracers. In neural applications, indirect metabolic or biomolecular activity can be recorded (that is, glucose or neurotransmitters), similar to the concept of MRI. Recent advancements, like optical and ultrasound imaging, have been the miniaturization of PET scanners for behavioral studies of freely moving mice (*229*).

In an attempt to generalize modalities, it is often challenging to bin one technique to one modality. For example, many electrical-based (non-electrophysiological) sensors or stimulators could be classified as their own modality (that is, thermal or biochemical). Biosensors can detect biomolecules via electrical or optical transduction or other means. Biomolecular stimulation (release of ions or molecules) can be performed through fluidics or through electrophoretic or electromechanical release. In this regard, the modality chosen for categorization can depend on what the chain of signal transduction looks like and is largely arbitrary. Optogenetic probes in the end require electrical control of light sources. Another prime example is the neural dust concept, which technically uses electrodes to transduce local activity (electrical) but whose signal would be read to an implanted transducer “wirelessly” by ultrasound. It is no surprise then that combining modalities in series (aiding the propagation of signal from cell to digital data or vice versa) has and will lead to some of the most intriguing interfacing tools for future neuroscience discoveries.

## COMBINING MODALITIES

Each modality described above brings its own advantages such that combining two or more modalities in parallel into a single experiment, diagnosis, or therapy provides an exciting path forward. Of the modalities discussed for stimulation and recording, many require physical probes or nodes to be placed in close proximity to neural tissue to achieve specificity and/or localization. Traditionally, each separate probe (that is, a fiber for optical interfacing or a microwire for electrical interfacing) required individual insertion and separate interfacing. Advancements in micro- and nanofabrication and materials processing techniques have not only pushed the limits on form factors but also allowed for two, even three, separate modalities to be combined on a single probe.

Fluidic or chemical delivery combined with electrical recording, for example, can allow the immediate and local effect of drug delivery on tissue response to be directly monitored. Early advances in this sense relied on fabrication schemes known from MEMS technology and wafer bonding to develop silicon probes with microfluidic channels (*230*). These approaches have been translated to, for example, SU-8, parylene, and elastomeric probes (*58*, *231*, *232*). However, the requirement of a microfluidic channel makes potential integration into ultrathin, <10-μm form factors challenging. The electrophoretic delivery device described in the “Active interfacing” section, the OEIP, can overcome this challenge and combine chemical delivery with electrical recording at subcellular size scales on thin substrates (*101*).

Simultaneous optical and electrical stimulation and recording have gained particular attention with the advent of optogenetics as a means to stimulate a predefined subset of neurons and electrically record the resulting electrophysiological response. For example, carbon-based electrode materials have been used to yield fully transparent ECoG grids through which optical stimulation and imaging can be performed (*38*, *233*). Another approach has been the integration of electrical recording sites along the shaft of an optical fiber, which found early utility (*234*). However, recent efforts have pushed for the light-guiding probe to serve a dual functionality, including recording electrical signals, for example, using the transparent semiconductor ZnO to simultaneously guide light and record potentials (*235*) or thermally drawing multifunctional fibers (as in Fig. 3E) (*55*). Monolithic integration of waveguides also presents a means by which light can be locally guided toward the vicinity of multiple electrical recording sites in both Si-based and polymer probes (*56*, *175*, *236*) (as in Fig. 3F). Rather than piping external light in, integrated micro-LEDs (see the “Hardware for optical stimulation and recording” section) can be patterned on multifunctional ultrathin probes; this approach can allow for integration of multiple light sources (with potentially different stimulation wavelengths) to be colocalized with photodetectors and electrical recording sites (*63*).

As components are downscaled, and creative fabrication methods are used, it is foreseeable that three or more modalities could be combined in a space-efficient and easily deployed fashion. The work of Canales *et al*. (*55*) with fibers and Rubehn *et al*. (*56*) with MEMS-based polymer probes provides two examples of the potential for combined optical, chemical (fluidic), and electrical bidirectional probes (*55*).

Finally, as a means to explore the effect of local stimulation on network- and organ-level activity, combining imaging techniques with local stimulators presents an exciting opportunity. One example is the combination of the magnetic modality, such as fMRI, with both optogenetic stimulation and electrical recording to investigate how optogenetic stimulation affects brain-wide activities (*237*). This method has been recently demonstrated as a valuable tool to study depression- and schizophrenia-related neural circuits in awake rats (*238*).

## OUTLOOK AND ROAD MAP

The efforts outlined above present the most recent in a broad set of neuroscience tools necessary to move treatments for brain disease forward: modalities that will enable long-term, minimally invasive, and widespread recording and stimulation of massive numbers of neurons, simultaneously. Work that decreases the neuroinflammatory response is especially important because understanding principles that underpin the rejection of implanted devices will inform future device form and may be applied to existing devices. An instructive parallel may be found in the vascular literature in the development of coronary artery stents, which progressed through many iterations over decades to overcome challenges with delivery, biological/nonbiological interfaces, and long-term function.

As the format and density of collected data grow, the questions of data extraction, handling, and analysis are brought to the fore. For example, at current levels of resolution and channel number, imaging of an entire mouse brain can reach the data range of 1 to 10 terabytes (*239*). Furthermore, collecting high-frequency, multisite, and multimodal data during long-term behavioral studies can further exacerbate the problem of data collection bandwidth and storage and becomes a challenge, especially as efforts to minimize the form factor of entire systems (including unwiring) continue. Power, data storage, and wireless transmission protocols and security are glaring areas of development required to indulge the desire to collect more while carrying less.

A separate challenge in the immense amount of exploratory work being carried out in biology and neuroscience is a lack of standardized experimental designs, or standardized reporting of experimental design, that prevents comparisons of results across data sets and that decreases reproducibility (*240*, *241*). The use of standardized, predetermined endpoints in biology is difficult because of natural biological variability but, even so, is commonly used in medicine; the European Union–funded Human Brain Project (*9*) aims to create just such a platform for sharing data in standardized formats.

Here, a number of modalities for both stimulation and recording are discussed. The merits of one modality over another depend on a number of factors: use/application, intended duration of interface, accessibility of target region, targeted cell type or size of population, and technological/clinical maturity. For example, are devices intended for clinical use or as research tools? Are they for short-term diagnosis/treatment or long-term implantation? The existing infrastructure and clinical acceptance for bidirectional electrical interfacing suggest that modifications in materials and form factors face fewer hurdles to implementation (with the exception of regulatory procedures); however, baring advancements in power handling and wireless data transmission, these tools require wires or controls using traditional electronic components. In addition, these tools require proximity of device for both recording and stimulation, for which stimulation is indiscriminate. Optical, magnetic, and other modalities face their own challenges in implementation, including downscaling of imaging tools, potential gene therapy, or injection of molecular/nanoparticular material. However, the promise of (parallel and complementary) cell-specific recording and stimulation is a key driver, especially for optogenetics. Many of the other cell-specific stimulation modalities (magnetic, ultrasound) are in their infancy, requiring significant efforts to understand their operating mechanism and efficacy. It is likely too soon to ask if one modality will “win” compared to the others, especially as the need or preference to combine multiple approaches is gaining interest.

Although many of the tools discussed here are meant for fundamental research, including mapping of neural circuits or testing possible mechanisms in progressions of diseases using model systems, a number of these tools ultimately seek clinical implementation for diagnostics and therapeutics. Whether it is a new material that is of interest, a probe architecture, or a molecular indicator dye or protein, the regulatory hurdles required for broad implementation seem insurmountable. Just the timeline for approval can slow and sometimes halt the iterative innovation cycle needed. For example, a new passive medical material used in a preexisting device can take 5 to 10 years to make it from bench to commercial medical device via FDA approvals (*242*). Nevertheless, routes to test new concepts exist, including the less stringent requirements for Institutional Review Board approvals for intraoperative studies (*67*), as well as creative routes to test deeper concepts, such as the combined use of viral delivery approaches and optogenetic tools to restore vision (identifier NCT02556736), which may inform future optogenetic implementation.

In working toward creating new medical and experimental devices, less invasive generally means more widely applicable. As an example, the number of patients with Parkinson’s disease in the 10 most populous countries is projected to double to 9 million by 2030 (*243*); the current deep-brain stimulation approach requires implantation of large electrodes, a neurosurgical operating room staff, intensive care unit admission, and close follow-up, and costs around $35,000 (*244*). A cortical surface, epidural, or even wearable device that accomplishes the same therapeutic endpoint would be able to help many more patients. In laying out constraints, it is important to consider that nominally equivalent tools that allow for reliable neural modulation at a distance will be more widely adopted than even the most neuroinflammatory-resistant, biocompatible implants.

With optogenetics alone, the ability to selectively modulate defined populations of neurons not only unlocks countless research opportunities but also has the potential to underpin an entirely new class of therapeutics. A vision of real-time neural activity detection, decoding, and modulation requires distributed, stable signal acquisition, miniaturized decoding hardware, and light delivery devices that escape neuroinflammatory surveillance but would be applicable to virtually any neurological disease.

The progression from Galvani’s stimulation of exposed frog leg nerve with charged metal implements to the capabilities of today is remarkable. However, much remains to be done; neuroscientists, material scientists, and physicians must continue to draw from other disciplines. Knowing how the central nervous system functions is a necessary precursor to a quantitative and concrete description of how neurological and psychiatric diseases give rise to behavioral and cognitive deficits. Here, we have described certain creative and diverse routes through which form factor and modality can be engineered to create tools designed to enable researchers and physicians to interrogate neural circuitry. The common link binding the successes of neuroscience in the past, and solving these hurdles in the future, is cross-disciplinary collaboration. These efforts are critical to success in the daunting, and exciting, problems that are within the grasp of neuroscience.
